# Risk Factors for Poor Outcome after Palliative Surgery for Metastatic Spinal Tumors

**DOI:** 10.3390/jcm12103442

**Published:** 2023-05-13

**Authors:** Akinobu Suzuki, Hidetomi Terai, Shinji Takahashi, Minori Kato, Hiromitsu Toyoda, Koji Tamai, Yusuke Hori, Yuki Okamura, Hiroaki Nakamura

**Affiliations:** Department of Orthopaedic Surgery, Graduate School of Medicine, Osaka Metropolitan University, Osaka 545-8585, Japan; terai@omu.ac.jp (H.T.); a99m042@yahoo.co.jp (S.T.); minori202048@gmail.com (M.K.); ocutoyoda@gmail.com (H.T.); koji.tamai.707@gmail.com (K.T.); yusukehori0702@gmail.com (Y.H.); yuki.muiminn.om@gmail.com (Y.O.); hnakamura@omu.ac.jp (H.N.)

**Keywords:** spinal metastasis, palliative surgery, activity of daily living, neurological function, ambulatory status, poor outcome, risk factors, hemoglobin, anemia, Tokuhashi score

## Abstract

Palliative surgery is performed to improve the quality of life of patients with spinal metastases. However, it is sometimes difficult to achieve the expected results because the patient’s condition, and risk factors related to poor outcomes have not been well elucidated. This study aimed to evaluate the functional outcomes and investigate the risk factors for poor outcomes after palliative surgery for spinal metastasis. We retrospectively reviewed the records of 117 consecutive patients who underwent palliative surgery for spinal metastases. Neurological and ambulatory statuses were evaluated pre- and post-operatively. Poor outcomes were defined as no improvement or deterioration in functional status or early mortality, and the related risk factors were analyzed using multivariate logistic regression analysis. The results showed neurological improvement in 48% and ambulatory improvement in 70% of the patients with preoperative impairment, whereas 18% of the patients showed poor outcomes. In the multivariate analysis, low hemoglobin levels and low revised Tokuhashi scores were identified as risk factors for poor outcomes. The present results suggest that anemia and low revised Tokuhashi scores are related not only to life expectancy but also to functional recovery after surgery. Treatment options should be carefully selected for the patients with these factors.

## 1. Introduction

With major advances in the treatment of cancer, the prognosis of patients with malignant tumors has improved [1], and the incidence of bone metastasis has also increased because the cumulative incidence increases over time after diagnosis [2]. The spine is the most common site for bone metastases [3]. Spinal metastasis causes pain and paralysis, leading to severe impairment in patients’ activities of daily living (ADL) and quality of life (QOL). Palliative surgery is an effective treatment for improving patients’ ADL and QOL. After Patchell’s report, which proved the efficacy of palliative surgery over radiation therapy [4], the number of surgeries have increased over time with the development of surgical methods and instruments [5,6]. Percutaneous vertebroplasty (VP)/balloon kyphoplasty (BKP) [7], decompression [8], spinal instrumentation [9], and a combination of these methods, with or without tumor resection [10,11], are standard options for palliative surgery. The indication and selection of surgical methods are determined by the invasiveness of the methods and patients’ life expectancy; however, it is sometimes difficult to achieve a good outcome due to their condition and/or rapid progression of primary cancer. Many studies have reported favorable surgical results [8,12,13]; however, the risk factors for poor outcomes have not been well studied.

Many factors associated with life expectancy in cancer patients have been reported for different types of cancer. Hemoglobin [14] and serum albumin [15] levels are associated with the prognosis of various cancers. Scoring systems are often used to predict the survival of patients with spinal metastases [16,17,18,19]. However, it remains unclear whether these factors influence functional outcomes after palliative surgery for spinal metastases, such as improvement in palsy or ADL.

In this study, we retrospectively analyzed the data of patients who underwent palliative surgery for spinal metastasis and investigated the risk factors for poor postoperative outcomes.

## 2. Patients and Methods

This was a retrospective study of consecutive patients who underwent palliative surgery for symptomatic spinal metastases at Osaka Metropolitan University Hospital between 2008 and 2021. In the treatment of symptomatic spinal metastasis in our institution, radiotherapy is considered for cases with (1) metastasis of a radiosensitive tumor, (2) pain without neurological deficits, (3) high risk for surgery or general anesthesia, and/or (4) prognosis <3 months; whereas palliative surgery is considered for cases with (1) progressive neurological deterioration, (2) spinal instability, (3) metastasis of a radioresistant tumor, (4) prognosis >3 months, (5) tolerable condition for general anesthesia or surgery, and/or (6) lasting symptoms despite previous radiotherapy to the lesion. All methods were performed in accordance with the Declaration of Helsinki and Ethical Guidelines for Medical and Health Research Involving Human Subjects in Japan. The study protocol was approved by our Institutional Review Board (No. 3170). Patients aged < 20 years and those who underwent curative surgery were excluded from this study. In total, 117 patients were included in the study. Data collected included primary tumor type, symptom duration before surgery, history of chemotherapy or radiation therapy before and after surgery, revised Tokuhashi score [17], spinal instability neoplastic score (SINS) [20], and American Society of Anesthesiologists physical status score [21]. Preoperative and postoperative ambulatory statuses were graded into five levels: ambulation without any support, with a T-cane, with a walker, wheelchair, and bedridden. The preoperative and postoperative palsy statuses were graded from A to E according to the Frankel classification [22]. The postoperative ambulation and palsy status were evaluated at discharge. Surgical data included surgical method (decompression only, vertebroplasty/balloon kyphoplasty (VP/BKP) only, decompression with VP/BKP, and stabilization with or without decompression), surgical time, and estimated blood loss during surgery.

In this study, poor outcome was defined as follows: (1) death during hospitalization for surgery; (2) no improvement in ambulatory status from the level of wheelchair or bedridden and no improvement in Frankel classification of A, B, or C; or (3) deterioration of ambulatory status or Frankel classification. If a patient had an ambulatory status of walker, T-cane, and without any support or a patient with Frankel D or E did not improve but maintained the status, the patient was not classified into the poor outcome group.

Each value was compared between the control and poor outcome groups using the chi-square test, Fisher’s exact test, or Mann–Whitney U test. Multivariate logistic regression analysis was performed to further investigate the factors related to poor outcomes, and odds ratios (ORs) with 95% confidence intervals (CIs) were calculated. Based on the results of the univariate analysis and clinical importance, multivariate models were adjusted for age, sex, ASA classification, symptom duration, total Tokuhashi score, hemoglobin level, and albumin level. The preoperative Frankel classification and ambulatory status were excluded because the Tokuhashi score included these factors. Instead, each element of the Tokuhashi score was further analyzed using univariate and multivariate logistic regression analyses adjusted for age, sex, ASA classification, and hemoglobin and albumin levels. All statistical analyses were performed using R software (version 3.5.1, Patched, http://www.r-proje ct.org accessed on 12 January 2023; R Foundation, Vienna, Austria). Statistical significance was set at *p* < 0.05.

## 3. Results

Demographic data are presented in Table 1 and Table 2. The mean age was 67.6 ± 9.3 years, and 79 patients (67.5%) were male. The lung was the most common primary site, followed by the kidneys, liver, prostate, and thyroid glands. Preoperatively, 88 patients (75.2%) were nonambulatory because of severe pain or paralysis, and 63 patients (53.8%) showed motor paralysis of Frankel A, B, or C. As a surgical procedure, decompression and stabilization with spinal instrumentation were the most common surgical procedures (69.1%), followed by VP/BKP (14.5%). No patient underwent separation surgery. For patients with spinal cord or nerve root compression without instability or massive osteolytic lesions, decompression alone (11.1%) or decompression with VP/BKP was performed. Emergency surgery was performed in 47 (40.2%) patients.

Of the 117 patients, 91 had neurological deficits before surgery, and improvement of one or more grades in the Frankel classification was observed in 44 patients (48.4%) (Table 3). Regarding ambulatory status, 95 patients could not walk without support preoperatively, and the ADL status improved postoperatively in 67 of the 95 patients (70.5%) (Table 4). Furthermore, 88 patients could not walk preoperatively; however, 47 (53.4%) patients could walk after surgery.

Finally, 21 patients (18.1%) were judged to have poor outcomes at discharge. A total of 7 patients died within 1 month of surgery (day 15, 2 patients; days 6, 10, 20, 24, and 26, 1 patient). In four patients, motor paralysis worsened at discharge (Frankel B to A, one patient; Frankel D to C, two patients; and Frankel E to D, one patient). In the other 11 patients, the status of motor paralysis remained unchanged after surgery (Frankel B, 1 patient; Frankel C, 6 patients; and Frankel E, 4 patients), and their activity worsened (wheelchair to bedridden, 2 patients) or remained nonambulatory without status improvement (bedridden to bedridden, 3 patients; wheelchair to wheelchair, 6 patients).

In the univariate analysis, the poor outcome group showed significantly lower total Tokuhashi scores (*p* < 0.01), hemoglobin levels (*p* < 0.01), and albumin levels (*p* = 0.048) than the good outcome group (Table 5). A significantly lower percentage of patients received chemotherapy or radiotherapy in the poor outcome group than in the good outcome group (28.5% vs. 63.5%, *p* < 0.01, and 14.3% vs. 41.7%, *p* < 0.05, respectively). In the poor outcome group, the ASA classification tended to be higher (*p* = 0.059), symptom duration tended to be shorter (*p* = 0.051), and the rate of nonambulatory patients tended to be higher (*p* = 0.096); however, these were not statistically significant. Multiple logistic regression analysis showed that a lower Tokuhashi score (OR 0.61, *p* < 0.01) and hemoglobin level (OR 0.5, *p* < 0.01) were significantly associated with poor outcomes (Table 6). As the Tokuhashi score contains multiple factors, we further analyzed the relationship between poor outcomes and each factor (Table 7). In the univariate analysis, the poor outcome group showed a significantly lower general condition (performance status [PS]) and more extraspinal and spinal bone metastases. Multivariate analysis adjusted for age, sex, ASA classification, hemoglobin level, and albumin level revealed that a lower general condition (OR 0.17, *p* < 0.01), a higher number of extraspinal bone metastases (OR 0.31, *p* < 0.01), a higher number of metastases in the vertebral body (OR 0.26, *p* < 0.01), and metastases to the major internal organs (OR 0.52, *p* = 0.03) were independent factors associated with poor outcomes.

### Case Presentation

**Figure 1:**
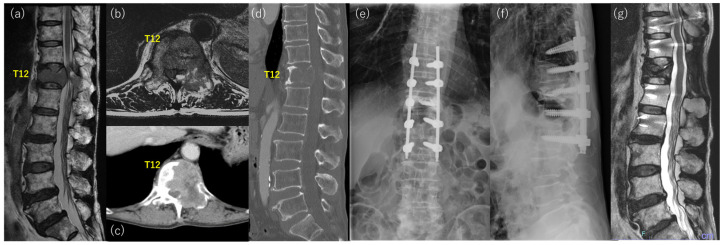
Representative case of the good outcome group with T12 spinal metastasis from lung adenocarcinoma. (**a**) sagittal image and (**b**) axial image of preoperative T2 weighted MRI, (**c**) axial image and (**d**) sagittal image of preoperative CT, (**e**) anterior-posterior and (**f**) lateral postoperative X-ray, (**g**) sagittal image of T2 weighted MRI at 3 years after surgery.

Case 1: A 69-year-old man with severe back pain and gait disturbance presented to our hospital for surgery (Figure 1). He had no history of malignancy, and the primary cancer was unknown at presentation. He was bedridden owing to severe back pain and presented with bowel and bladder dysfunction. Physical examination revealed hypesthesia and motor weakness in the lower extremities. The total Tokuhashi score was 5 points, and the SINS score was 10 points. Imaging studies showed osteolytic change with vertebral collapse at T12 and severe compression of the spinal cord (Figure 1a–d). Decompression between T11 to L1, T12 vertebroplasty, and percutaneous fixation from T10 to L2 were performed, and radiation therapy (30 Gy/10 Fr) was also performed after surgery (Figure 1e,f). Finally, the patient was diagnosed with lung adenocarcinoma based on histological examination and other imaging studies. He could walk without any aid, and he was discharged 40 days after surgery. He was treated with an immune checkpoint inhibitor, and a complete response was obtained. The T12 lesion exhibited shrinkage and osteosclerotic changes (Figure 1g). The patient survived and was ambulatory for more than 5 years after surgery.

**Figure 2:**
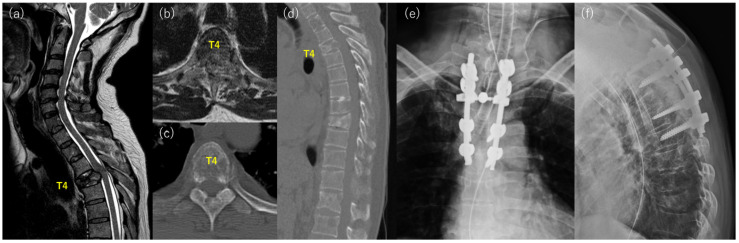
Representative case of the poor outcome group with T4 spinal metastasis from lung adenocarcinoma. (**a**) sagittal image and (**b**) axial image of preoperative T2 weighted MRI, (**c**) axial image and (**d**) sagittal image of preoperative CT, (**e**) anterior-posterior, and (**f**) lateral postoperative X-ray.

Case 2: A 60-year-old man with back pain was referred to our hospital for further examination and treatment (Figure 2). Imaging studies showed multiple metastases of the spine, and vertebral collapse and spinal cord compression were found at T4 (Figure 2a–d). He had no history of malignancy, but a biopsy of the spinal lesion and the imaging study revealed lung adenocarcinoma. He could walk without neurological deficits at presentation, and radiation therapy was initiated. However, motor weakness progressed during this period, and he underwent posterior decompression with stabilization from T2 to T6 (Figure 2e,f). The motor weakness improved immediately after surgery. However, respiratory status progressively worsened due to exacerbation of primary cancer, and the patient died 10 days after the surgery.

## 4. Discussion

In this study, we retrospectively analyzed the preoperative and postoperative data of patients with spinal metastases who underwent palliative surgery. The results showed that functional recovery or maintenance of the ability to walk was obtained in 82% of the patients, whereas the remaining 18% showed poor outcomes, with no improvement or deterioration of ADL/neurological status or early death. Low revised Tokuhashi score, and low hemoglobin level were identified as risk factors for poor outcomes. Although this study was not an analysis of a large sample size, it is the first study to investigate the risk factors for poor surgical outcomes on function in detail, including blood examination.

Many studies have demonstrated that surgical treatment is effective in improving ADL and/or QOL in patients with spinal metastases [4,23,24,25,26]. Neurological function was evaluated using the Frankel classification or the American Spinal Cord Injury Association impairment scale in most studies. Choi et al. [24] conducted a large-scale prospective longitudinal study of patients who underwent surgery for spinal metastasis and reported that 45% of the patients with Frankel scores of A through C improved to the level of walking with assistance or independently (Frankel D and E) after surgery. Barzilai et al. [25] reported that 53% of patients with neurological deficits improved by one or more grades of Frankel classification 6 weeks after surgery. In our study, an improvement in Frankel grade was observed in 48% of patients with preoperative neurological deficits, and the results were comparable with those of previous studies. However, various methods have been used to evaluate ADL. Liu et al. [26] conducted a systematic review and meta-analysis and reported that the mean recovery of ambulation in nonambulatory patients preoperatively was 27.4%. Kanda et al. [23] demonstrated that the PS, Barthel index (BI), and EQ-5D improved in almost 90% of patients after surgery; however, re-deterioration after the initial improvement was observed in PS (15.6%), BI (14.3%), and EQ-5D (18.8%). We believe that recovery from bedridden to wheelchair conditions remains beneficial for patients, although both are classified as nonambulatory. On the other hand, BI is relatively detailed, and a one-grade improvement of one item may not be meaningful to patients. Thus, we classified ambulatory/mobility status into five grades in the present study, and 70.5% of the patients with impaired mobility recovered after surgery. However, early death, deterioration, or no improvement in low functional status was observed in 18% of the patients. It is difficult to clearly define poor outcomes; nonetheless, we defined them in the present study because the objectives of the surgery were not achieved in these patients.

We also included death during hospitalization for surgery in the poor outcome group, and seven patients showed early mortality. Six patients died due to exacerbation of the primary cancer (respiratory failure, four cases; brain metastasis, one case; carcinomatous lymphangiosis, one case), and one patient died by suicide. Two patients underwent vertebroplasty because of the predicted short prognosis, and the remaining four patients underwent posterior decompression fusion because the prognosis was not judged to be short. There was no complication leading to mortality in these cases; surgical invasion might cause rapid progression of the primary cancer. It is sometimes difficult to predict such early mortality in patients with spinal metastasis, especially of unknown origin. The previously reported scoring system [17,18,27] and the results of the present study will help in predicting the prognosis. However, it is also important to preoperatively explain the possibility of rapid exacerbation of primary cancer in the early period after surgery.

Postoperatively, a significantly lower percentage of the patients received systemic anti-cancer drug therapy in the poor outcome group than in the good outcome group (28.5% vs. 63.5%, *p* < 0.01). Recent advances in targeted therapy and immunotherapy for specific gene mutations or alterations dramatically improve the treatment outcome of malignancy [28,29] (Figure 1). Furthermore, the methods for patient selection have almost been established, and the efficiency of these novel therapies is being improved [30]. Although we could not analyze the long-term outcomes and prognosis after the surgery, the good outcomes, even in the short term, may create a chance to receive those targeted therapies, resulting in prolonged good outcomes and prognosis.

The original [31] and revised [17] Tokuhashi scores are systems designed to determine prognosis. Although some modifications are required with the advent of molecular-targeted drugs and immune checkpoint inhibitors [32], their validity has been proven by many studies [16,33], and they remained widely used for prognostic purposes. In the present study, the revised Tokuhashi scores were strongly associated with poor functional outcomes. Several reports have demonstrated that the Tokuhashi score is related not only to life expectancy but also to functional recovery [34,35,36]. Yamashita et al. [34] reported that the preoperative low Tokuhashi score group (0–8 points) showed significantly lower PS improvement than the high Tokuhashi score group (9–15 points). Ohashi et al. [36] demonstrated that a Tokuhashi score < 8 points is an independent risk factor for failure to regain ambulatory ability after surgery. The Tokuhashi score includes items related to performance and neurological status. Previous reports have also indicated that preoperative performance status and neurological function influence postoperative physical function [24,36], which may be one of the reasons why the Tokuhashi score was identified as a factor associated with poor outcomes. In contrast, the multivariate analysis of each domain showed that the number of extraspinal bone metastases, the number of metastases in the vertebral body, and metastases to major internal organs were also factors involved in the postoperative outcome. Even if the problems in the responsible spinal lesion are resolved by surgery, the patient’s ADL may not improve as expected due to symptoms of other bony lesions or deterioration of the general condition with worsening internal organ lesions.

Several mechanisms, such as bone marrow involvement, tumor-associated blood loss, elevation of inflammatory cytokines, and iron or folic acid deficiencies, cause anemia in patients with cancer [37], and anemia has been proven to affect the prognosis of many types of cancer [14,38,39]. However, few studies have demonstrated the relationship between anemia and prognosis in patients with spinal metastases. For instance, Yang et al. [40] conducted a retrospective and prospective cohort study in which anemia was identified as a significant prognostic factor in patients with spinal metastases of unknown primary origin. To the best of our knowledge, this is the first study to demonstrate that hemoglobin levels are associated with functional recovery after surgery for spinal metastases. Although early mortality may have influenced the analysis results because it was included in the poor outcome group, the early mortality in the poor outcome group was only 28%. Therefore, anemia is likely to have adverse effects on postoperative functional recovery. Although the reasons for this were not clarified in the present study, several hypotheses can be considered. Tumor progression induced by hypoxia can be intensified by anemia [41], which may affect a patient’s general condition after surgery. Fatigue or low activity related to anemia [42] may impede postoperative rehabilitation, and the predicted improvement may not be achieved. Nonetheless, further studies are required to clarify the mechanism by which anemia impairs functional recovery.

This study has several limitations. First, this was a retrospective study, and the study population was heterogeneous and relatively small. Patients with various primary cancers were included in different proportions, and several surgical procedures were performed in different situations. Therefore, a large-scale prospective multicenter study is necessary to elucidate the impact of primary cancer and surgical methods on functional outcomes with decreasing biases. Second, we evaluated the outcome at discharge but not at a certain time point after surgery. This is because early recovery is ideal and necessary for patients with cancer, and the evaluation is accurate without loss to follow-up. Lastly, we could not analyze the patients’ survival. As many patients were transferred to another convalescent hospital after surgery, we could not obtain data about the survival of all patients. Future studies are needed to confirm whether the risk factors identified in this study are related to long-term functioning or survival.

## 5. Conclusions

In conclusion, palliative surgery for spinal metastases is effective for the improvement or maintenance of patient function in most patients. Our data showed that 18% of patients exhibited poor outcomes, and low hemoglobin levels and low revised Tokuhashi scores were identified as risk factors for poor recovery after palliative surgery. Surgical treatment should be performed cautiously in patients with these factors.

## Figures and Tables

**Table 1:** Demographic data.

	*n* = 117
Age, year	67.6 ± 9.3
Male sex, *n* (%)	79 (67.5)
Mean Tokuhashi score	7.2 ± 3.0
Mean SINS	10.5 ± 2.7
ASA-PS, *n* (%)	
2	83 (70.9)
3	30 (25.6)
4	4 (3.4)
Symptom duration, days	88.1 ± 112.6
Preoperative ambulatory/mobility status, *n* (%)	
Without support	22 (18.8)
Cane	5 (4.3)
Walker	2 (1.7)
Wheelchair	42 (35.9)
Bedridden	46 (39.3)
Preoperative Frankel classification, *n* (%)	
A	1 (0.9)
B	10 (8.5)
C	52 (44.4)
D	28 (23.9)
E	26 (22.2)
History of chemotherapy, *n* (%)	45 (38.5)
History of radiation therapy, *n* (%)	34 (29.1)
Preoperative level of hemoglobin, g/dL	12.2 ± 1.8
Preoperative level of serum albumin, g/dL	3.6 ± 0.5
Emergency operation, *n* (%)	47 (40.2)
Surgical methods, *n* (%)	
Decompression	13 (11.1)
VP/BKP	17 (14.5)
Decompression with VP/BKP	6 (5.1)
Decompression and stabilization	81 (69.1)
Operating time, min	198.6 ± 107.7
Blood loss, mL	667.8 ± 951.4
Length of hospital stay, day	28.9 ± 23.4
Postoperative chemotherapy, *n* (%)	67 (57.3)
Postoperative radiotherapy, *n* (%)	43 (36.8)

SINS, Spinal Instability Neoplastic Score; ASA-PS, American Society of Anesthesiologists Physical Status; VP, vertebroplasty; BKP, balloon kyphoplasty

**Table 2:** Distribution of primary malignant tumor.

Primary Malignant Tumor	*n* (%)
Lung	21 (17.9)
Kidney	20 (17.1)
Liver	12 (10.3)
Prostate	12 (10.3)
Thyroid	10 (8.5)
Malignant lymphoma	8 (6.8)
Esophagus/stomach/intestine	8 (6.8)
Sarcoma	7 (6.0)
Urothelium	7 (6.0)
Breast	4 (3.4)
Multiple myeloma	3 (2.6)
Esophagus	3 (2.6)
Pancreas	2 (1.7)
Others	3 (2.6)

**Table 3:** Pre- and post-operative Frankel classification.

		Postoperative					
Preoperative	No. of Cases	A	B	C	D	E	Died
A	1	0	0	0	0	0	1
B	10	1	1	5	2	0	1
C	52	1	1	17	32	1	0
D	28	0	0	3	19	4	2
E	26	0	0	0	1	25	0

**Table 4:** Pre- and post-operative ambulatory status.

		Postoperative					
Preoperative	No. of Cases	Bedridden	Wheelchair	Walker	Cane	Without Support	Died
Bedridden	46	4	17	7	5	8	5
Wheelchair	42	2	12	16	10	1	1
Walker	2	0	0	1	0	1	0
Cane	5	0	0	0	4	1	0
Without support	22	1	0	0	0	20	1

**Table 5:** Univariate analysis of factors.

	Good Outcome	Poor Outcome	*p*-Value
*n*	96	21	
Age, year	67.5 ± 9.3	68.0 ± 9.4	0.801
Male sex, *n* (%)	63 (65.6)	16 (76.2)	0.445
ASA-PS			
2	72 (75.0)	11 (52.4)	0.059
3	22 (22.9)	8 (38.1)	
4	2 (2.1)	2 (9.5)	
Symptom duration, days	96.2 ± 121.7	51.0 ± 45.5	0.051
Mean Tokuhashi score	7.6 ± 2.8	5.1 ± 2.9	<0.01
Mean SINS	10.4 ± 2.7	11.2 ± 2.7	0.157
Preoperative ambulatory status, *n* (%)			
Without support	20 (20.8)	2 (9.5)	0.575
Cane	5 (5.2)	0 (0)	
Walker	2 (2.1)	0 (0)	
Wheelchair	32 (33.3)	10 (47.6)	
Bedridden	37 (36.5)	9 (42.9)	
Preoperative Frankel classification, *n* (%)			
A	0 (0)	1 (4.8)	0.264
B	7 (7.3)	3 (14.3)	
C	44 (45.8)	8 (38.1)	
D	24 (25.0)	4 (19.0)	
E	21 (21.9)	5 (23.8)	
Preoperative level of hemoglobin, g/dL	12.4 ± 1.8	11.0 ± 1.6	<0.01
Preoperative level of serum albumin, g/dL	3.6 ± 0.49	3.4 ± 0.50	0.048
Emergency operation, *n* (%)	39 (40.6)	8 (38.1)	1
History of chemotherapy, *n* (%)	34 (35.4)	11 (52.4)	0.215
History of radiation therapy, *n* (%)	29 (30.2)	5 (23.8)	0.791
Surgical methods, *n* (%)			
Decompression	10 (10.4)	3 (14.3)	0.144
VP/BKP	11 (11.5)	6 (28.6)	
Decompression with VP/BKP	5 (5.2)	1 (4.8)	
Decompression and stabilization	70 (72.9)	11 (52.4)	
Postoperative chemotherapy, *n* (%)	61 (63.5)	6 (28.6)	0.023
Postoperative radiotherapy, *n* (%)	40 (41.7)	3 (14.3)	<0.01

SINS, Spinal Instability Neoplastic Score; ASA-PS, American Society of Anesthesiologists Physical Status; VP, vertebroplasty; BKP, balloon kyphoplasty.

**Table 6:** Multivariate logistic regression analysis of factors.

Factors	OR	95% CI	*p*-Value
Age	1.01	0.94–1.08	0.88
Sex	2.84	0.65–12.4	0.166
ASA	2.11	0.77–5.83	0.148
Symptom duration	0.99	0.98–1.00	0.255
Surgical Method	1.77	0.72–4.38	0.215
Tokuhashi score	0.62	0.47–0.81	<0.01
Hb	0.5	0.32–0.76	<0.01
Alb	1.47	0.31–7.02	0.632

OR, odds ratio; CI, confidence interval; ASA, American Society of Anesthesiologists; Hb, hemoglobin; Alb, albumin.

**Table 7:** Univariate and multivariate logistic regression analyses for each element of the Tokuhashi score.

	Univariate			Multivariate		
	OR	95% CI	*p*-Value	OR	95% CI	*p*-Value
General condition	0.23	0.086–0.6	<0.01	0.19	0.06–0.60	<0.01
No. of extraspinal bone metastases	0.42	0.23–0.76	<0.01	0.31	0.15–0.67	<0.01
No. of vertebral body metastases	0.44	0.23–0.82	0.01	0.28	0.12–0.64	<0.01
Metastases to major internal organs	0.67	0.41–1.11	0.123	0.52	0.28–0.94	0.03
Primary site of cancer	0.92	0.71–1.19	0.51	0.97	0.71–1.31	0.82
Palsy	0.65	0.28–1.5	0.31	0.46	0.16–1.29	0.14

OR, odds ratio; CI, confidence interval.

## Data Availability

The data that support the findings of this study are available from the corresponding author, A.S., upon reasonable request.
